# Nutritional management practices and perspectives of Radiation Oncologists: outcomes of an International Atomic Energy Agency survey

**DOI:** 10.1016/j.ctro.2026.101196

**Published:** 2026-05-25

**Authors:** Alexia J. Murphy-Alford, Jonathan P. Bennett, Aleksandra Elwertowska, Elena Fidarova, Aaron Grossberg, Deepa Puttaswamy, Nuzhat Zahin, Yavuz Anacak

**Affiliations:** a Nutritional and Health-Related Environmental Studies Section, Department of Nuclear Sciences and Applications, International Atomic Energy Agency, Vienna, Austria; b Department of Epidemiology, University of Hawai’i Cancer Center, Honolulu, HI, USA; c Applied Radiation Biology and Radiotherapy Section, Department of Nuclear Sciences and Applications, International Atomic Energy Agency, Vienna, Austria; d Cancer Early Detection Advanced Research Center (CEDAR), Knight Cancer Institute, Oregon Health and Science University, Portland, OR, USA; e Brenden-Colson Center for Pancreatic Care, Oregon Health and Science University, Portland, OR, USA; f Department of Radiation Medicine, Oregon Health and Science University, Portland, OR, USA; g Division of Nutrition, St. John’s Research Institute, St. John’s National Academy of Health Sciences, Bengaluru, Karnataka, India; h Department of Radiation Oncology, Ege University Faculty of Medicine, Izmir, Turkey

**Keywords:** Radiotherapy, Nutrition therapy, Malnutrition, Body composition, Global oncology

## Abstract

**Background:**

Radiation therapy (RT) commonly leads to nutrition‑related adverse effects. Poor nutritional status is associated with worse outcomes, yet nutrition support remains underprioritized. This study explores Radiation Oncologists (ROs) perspectives and practices in nutrition management in RT settings across diverse global contexts.

**Methods:**

An electronic survey developed by the IAEA was distributed globally through a mailing list and social media. The questionnaire comprised 38 items on hospital practices and professional perspectives. Data were analysed using descriptive statistics, Chi-squared or Fisher’s exact tests and post‑hoc pairwise comparisons.

**Results:**

The survey respondents included 209 ROs from 65 countries. Most participants (81.6%) regarded nutrition as extremely important in RT, and 93.8% believed it influences clinical outcomes. Common symptoms reported were weight loss, mucositis, dysphagia, loss of appetite, nausea, and taste changes. Barriers to nutrition care included financial constraints, lack of access to nutrition tools, and dietitian time constraints, varying across country income groups. Only 42.2% of hospitals had a dietitian dedicated to oncology, and routine dietetic consultation for patients undergoing RT only occurred in 26.5% of hospitals. Weight and clinical judgement were the most used indicators of nutritional status and only 18.2% of hospitals assessed body composition in patients undergoing RT.

**Conclusion:**

Nutrition support is recognized as essential in RT, yet significant gaps remain in workforce capacity, resources, and utilisations of body composition assessments. Coordinated international efforts to integrate nutrition into routine cancer care are critical to improving patient outcomes.

## Highlights

• Nutrition is recognised as a critical component of RT support.
• Global survey reveals major gaps in nutrition care across RT centres.
• Global support is needed for oncology nutrition training and resources.

## Introduction

Cancer remains one of the leading causes of morbidity and mortality worldwide, and radiation therapy (RT) is central to its management, with approximately half of all patients requiring RT during their disease [1]. Malnutrition is a prevalent and often under-recognized complication among cancer patients, including those undergoing RT [2], [3]. Individuals with head and neck, oesophageal, and gastrointestinal cancers are particularly vulnerable to the side effects of RT on structures essential for chewing, swallowing, and digestion [4]. These effects frequently manifest as mucositis, dysphagia, anorexia, nausea, vomiting, and taste alterations, which can significantly impair oral intake and compromise nutritional status [5], [6]. Local toxicities are further compounded by systemic side effects such as fatigue, inflammation, and metabolic alterations that increase energy requirements while simultaneously reducing intake.

Numerous studies have underscored the strong association between nutritional status and clinical outcomes in patients with cancer. Poor nutritional status has been linked to treatment interruptions, higher infection rates, reduced treatment efficacy, prolonged hospital stays, diminished quality of life, and poorer survival [7], [8], [9], [10], [11]. Despite this evidence, nutritional care in radiation oncology remains inconsistently integrated into routine practice. While international guidelines are available, implementation of the recommendations varies widely across oncology institutions and healthcare systems [4], [12], [13], [14]. Effective nutrition management often requires a multidisciplinary approach, but the degree of collaboration and resource allocation differs substantially [15], [16], [17], [18], [19]. Patient-related factors such as socioeconomic status, cultural dietary preferences, and health literacy further complicate the delivery of tailored nutritional support [20], [21].

To promote the sustainable integration of nutritional care into RT services, understanding how nutrition care is perceived and currently implemented in diverse RT contexts is key. Little is known about how nutrition care practices and Radiation Oncologist (RO) perceptions differ across countries with varying resource levels, or how these differences affect the delivery of nutritional support during treatment. This study aimed to explore RO views on the importance of nutrition during RT and to describe current practices and perceived barriers to effective nutritional management within this clinical context.

## Methods

To understand the practices, perspectives, resources and challenges in providing nutrition care to cancer patients receiving RT, a survey was developed to gather insights from RO and other health professions who work with patients receiving RT. The survey was developed by the Nutritional & Health-related Environmental Studies (NAHRES) and Applied Radiation Biology and Radiotherapy (ARBR) Sections of the IAEA and was reviewed and piloted by a multidisciplinary team of ROs, dietitians and researchers. It was distributed through the ARBR mailing list and through LinkedIn, with colleagues requested to share widely with medical professionals in the RT field. The survey included 38 short answer and multiple-choice questions on nutrition practice at the hospital and the health professional’s perspective of nutrition care in RT patients. The questionnaire was established on Microsoft Forms and remained open for 60 days between March and May 2025.

### Data analysis

Descriptive statistics of only RO survey respondents were reported in this analysis. Analyses were conducted at two levels: respondent level analyses, where denominators reflect the number of ROs who answered each question, and hospital level analyses, where responses were grouped by institution using respondent reported hospital name and country. Duplicate institutional entries were identified by matching hospital name and country. Only the first fully completed response from each institution was included in hospital level analyses to avoid overrepresentation, and examination of submission order showed no systematic differences between early and later respondents, indicating that this approach was unlikely to introduce bias. World Bank 2024 Classifications based on GNI per capita were used to classify country income into three categories; high income (HIC), Upper Middle Income (UMIC) and combined Lower Middle Income and Low Income (LMIC-LIC) [22]. All data analysis was performed using R version 4.50 statistical software. Standard descriptive statistics were used to characterize the study population. Chi-squared or Fisher’s exact tests were used to determine whether there was an association between the income group, and one of the dependent variables of interest. If all expected counts were ≥5, Chi-squared was used, otherwise Fisher’s exact test was used. Values with missing dependent variable values were excluded from the analysis. Post-hoc pairwise comparison, with Holm correction for multiple comparisons, was performed for associations found to be statistically significant. Two-sided significance level was set to α = 0.05.

## Results

**Fig. 1:**
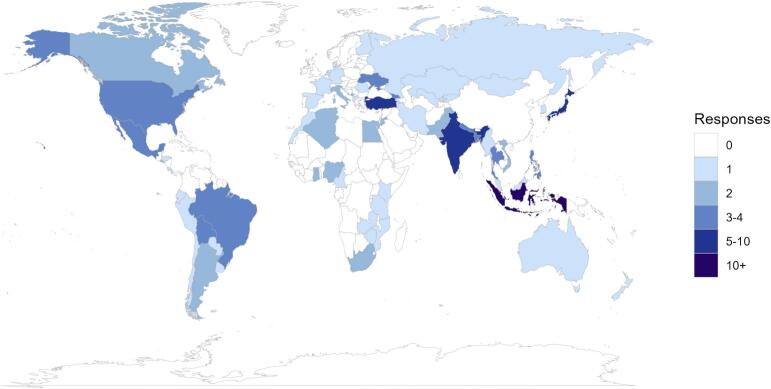
Countries represented in the survey (n = 65).

The survey was completed by 209 ROs. In total, 65 countries were represented in the survey (Fig. 1). Out of all respondents (n = 209), 56% were from UMIC (n = 116), 28% of respondents were from LMIC-LIC (n = 59), and 16% from high income countries (HIC) (n = 34) There were responses from 181 hospitals; 47% were public or government hospitals, 29% were private hospitals and 24% were classified as other types of hospitals.

### Radiation Oncologists' nutrition management perspectives

The survey revealed that 81.6% of 209 ROs regarded nutritional health of RT patients as extremely important, while 16.4% considered it somewhat important, 1.4% remained neutral and 0.6% believed it not important. While there was no significant difference between the perspectives of ROs in UMIC (82.5%) and those in HIC (64.7%) (p = 0.10), ROs in LMIC-LIC (89.8%) were significantly more likely to report that nutrition is extremely important than those in HIC (p = 0.02). Based on their clinical experience, 93.8% of ROs believed that nutritional status significantly influences clinical outcomes in patients receiving RT, this did not differ between income groups.

**Fig. 2:**
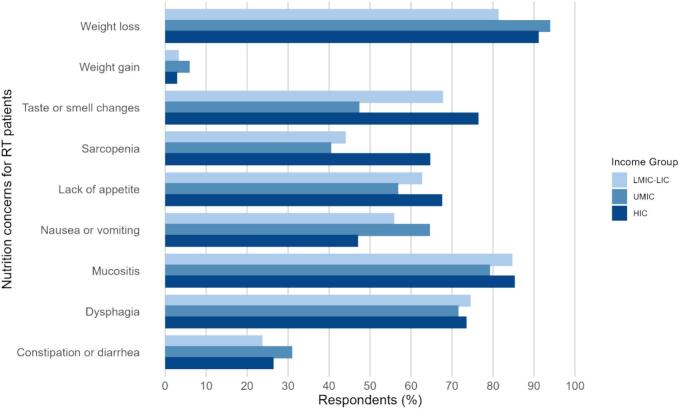
Nutrition symptoms reported by Radiation Oncologists' for patients receiving RT.

When asked which cancer patient population they believed experiences the highest incidence of nutrition-related toxicities during RT, from 208 responses, 90.0% identified Head and Neck as the most affected, followed by GI cancer patients (4.8%). ROs identified six nutrition symptoms frequently seen in cancer patients, including weight loss (90.0%), mucositis (81.8%), odynophagia/dysphagia (73%), lack of appetite (60.3%), nausea and vomiting (59.3%) and taste and smell changes (57.9%). Fig. 2 shows nutrition symptoms reported by income group. Taste and smell were reported as more common by ROs in HIC (76.5%) than UMIC (67.8%, p = 0.01), which was the only significant difference in nutrition symptoms by income.

**Fig. 3:**
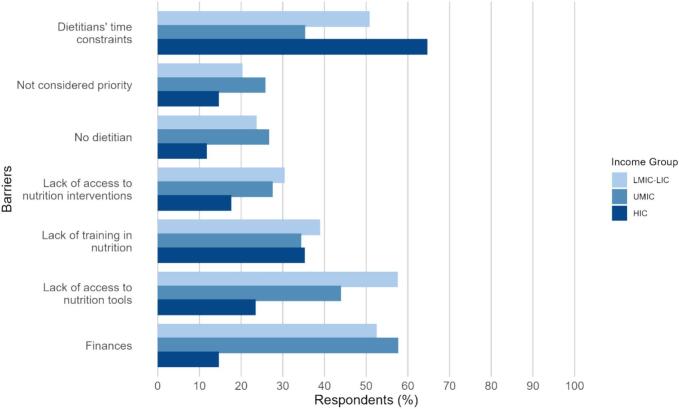
Barriers to delivery of effective nutritional services.

Based on answers from 209 respondents, the key barriers to the delivery of effective nutritional services identified were hospital-level financial constraints (49.3%), lack of access to nutrition tools (44.5%), and dietitians/nutritionists time constraints (44.5%). Fig. 3 displays barriers to providing effective nutrition services by income group. Time constraints were a significantly higher concern in HIC (64.7%) than UMIC (35.3%; p = 0.01) and there was no significant difference between HIC and LMIC-LIC (50.8%; p = 0.28). Finances were a significantly higher barrier in LMIC-LIC (52.5%; p = 0.001) and UMIC (57.8%; p = 0.001), than HIC (14.7%). Lack of access to nutrition tools as a barrier to providing nutrition services was significantly more of a concern in LMIC-LIC (57.6%), compared to HIC (23.5%; p = 0.009). There were no significant differences between income groups reported for the other barriers.

From 206 ROs, 48.5% reported that understanding body composition changes in patients receiving RT was extremely important; this did not vary significantly across income group. The most reported barriers to assessing body composition included limited access to appropriate equipment (55.7%), insufficient staff training (52.2%), financial constraints (51.7%), and lack of standardized protocols (49.5%). Perceived barriers did not differ significantly between income groups.

### Nutrition management practices

Responses were received from ROs representing 181 hospitals. Dietitians were available in 85.7% of hospitals, while dedicated oncology dietitians were only available in 42.2%. Among HIC, 84.6% of hospitals had at least one dedicated oncology dietitian, which was significantly higher than UMIC (51.5%; p = 0.014); the difference between HIC and LMIC-LIC (56.2%) was not statistically significant (p = 0.055). In only 26.5% of hospitals, a consultation with a dietitian is standard of care for patients undergoing RT.

When evaluating nutrition interventions provided to RT patients, only 43.3% of 164 hospitals who answered this question had nutrition education guidelines specifically for patients receiving RT, this did not differ significantly by income group. Out of 180 hospitals, 89.4% had access to nutrition counselling, 78.3% of hospitals had access to oral nutritional supplements, 66.7% had access to enteral nutrition, 61.7% had access to parental nutrition and 43.9% had access to appetite stimulants; these were not significantly different between income groups. When ROs were asked which nutrition interventions were most prescribed to patient undergoing RT at their hospital (n = 178), oral supplements (75.3%) and nutrition counselling (71.3%) were most common, followed by enteral nutrition (51.7%), appetite stimulants (33.1%), parenteral nutrition (32.0%) and traditional complementary or alternative medicine (12.4%); these did not significantly differ by income group.

**Fig. 4:**
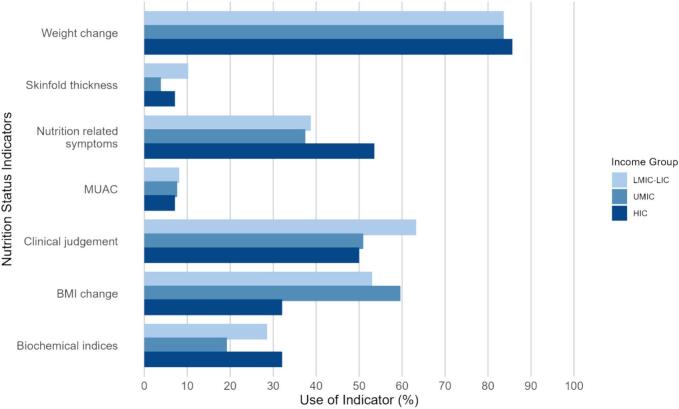
Indicators used to monitor nutritional status.

Of those who responded (n = 138), only 38.4% of hospitals undertook nutrition screening; this did not significantly differ by income group. When ROs were asked which indicators were used to monitor nutritional status in patients undergoing RT, 89.4% used weight, 57.6% used clinical judgement, 57.1% used BMI, 42.9% used nutrition related symptoms and 25.3% used biochemical indices (n = 170 responses). Skinfolds (6.5%) and Mid Upper Arm Circumference; (MUAC, 8.2%) were not often used. The indictors used to monitor nutritional status were not significant between income groups (Fig. 4). Of 148 hospitals, only 18.2% reported that they measure body composition in patients undergoing RT at their hospital; the responses were not significantly different between income levels. In the 27 hospitals that did measure body composition, the most used was bioelectrical impedance analysis (BIA; 55.6%), with only 18.5% using dual-energy X-ray absorptiometry (DXA).

As one UMIC contributed 54% of all UMIC responses, a sensitivity analysis was performed to assess whether this overrepresentation influenced the findings. All analyses comparing income groups were repeated after excluding responses from this country. The direction and statistical significance of the results remained unchanged, indicating that the observed patterns were not driven by disproportionate representation from any single country.

## Discussion

The findings of this study underscore the strong recognition among ROs of the critical role nutrition plays in the care of cancer patients undergoing RT. A large majority of respondents (81.6%) considered nutritional health to be extremely important. In previous studies in Saudi Arabia [23] and the UK [24], 65% of medical professionals rated nutrition as a crucial component of oncology care. ROs in LMIC and LIC were significantly more likely to consider nutrition to be extremely important, this may be due to patients frequently presenting with advanced disease and preexisting malnutrition [25]. In this study, 93.8% of ROs affirmed that nutritional status has a significant impact on clinical outcomes, similar to a study in Türkiye, were 94.5% of oncologists reported that nutritional status influences prognosis [26].

In this study, more than half of surveyed hospitals reported no dedicated oncology dietitian, and that the time constraints of the available dietitians were a major barrier to nutrition services. In most hospitals, dietitians were consulted only upon referral, and just one quarter have a nutrition consult as standard of care for RT patients. While clinical nutrition professionals should remain the principal providers of nutrition care, ROs can also possess a foundational understanding to support patients in resource limited settings. However, such training is not routinely incorporated into oncology education; for example, only 43% of ROs in Türkiye reported receiving nutrition training [23]. Globally, limited staff knowledge of nutrition has been identified as a persistent barrier in oncology practice [26], [27], [28] and the demand for nutrition education among oncology professionals is substantial; 90% of physicians in France [29] and 98.6% of Italian health professionals [16] reported strong interest in advanced training in clinical nutrition. Addressing these gaps requires investment both in expanding the presence of clinical nutrition professionals within RT settings and in developing structured, accessible, and context specific training programmes to strengthen ROs’ capacity to deliver nutritional care.

Patient-directed nutrition education should also be a cornerstone of supportive oncology care, yet such resources remain scarce in many RT settings. The lack of standardized, culturally appropriate, and easily comprehensible materials limits patients’ ability to engage in self-management. In this survey, less than half of hospitals reported having nutrition education guidelines for patients receiving RT. The routine availability of quality nutrition education resources for patients may represent a cost and time effective initial strategy for enhancing the nutritional care of individuals with cancer in low-resource settings. In these contexts, empowering patients through structured education materials can help mitigate the impact of limited clinical resources, promote earlier recognition of nutritional risk, and support more sustainable models of care.

The implementation of established nutrition guidelines and standardized screening tools within RT practice presents a critical opportunity to address persistent gaps in nutrition care. Lack of access to nutrition screening and assessment tools were a major barrier reported in UMIC and LMIC-LIC. In this study, over 60% of hospitals reported not using a nutrition screening tool for RT patients, and the absence of standardized nutrition tools were identified as a major barrier to delivering effective nutrition care. These findings underscore that, although evidence-based guidelines and screening tools are available [30], their translation from research into routine clinical practice within RT settings, while also accounting for variation in resources across countries and healthcare contexts [31] remains limited.

While nutrition screening identifies patients at risk, comprehensive nutrition assessment is essential to guide individualized interventions and optimize cancer treatment outcomes in RT patients. Most institutions surveyed relied on basic indicators such as weight and clinical judgement for nutrition assessment, with only 18.2% assessing body composition in patients undergoing RT. Among the small number of institutions where body composition assessments were performed, BIA was the most frequently employed modality, whereas DXA and CT were seldom utilized. Routine planning CT scans offer a practical avenue for opportunistic assessment of skeletal muscle, and DXA measurements similiarly enable evaluation of body composition alongside bone density. Integrating these measures into toxicity prediction models and adaptive RT workflows could facilitate earlier identification of highrisk patients and strengthen personalized nutritional management.

Based on the gaps identified in this global survey, we propose a set of practical, resource-stratified recommendations to support the integration of nutrition care into RT services (Table 1). These recommendations are intended to guide institutions, professional societies, and policymakers in strengthening nutrition pathways across diverse healthcare settings.

**Table 1:** Recommendations to support integration of nutrition care into RT services.

Domain	Recommended Actions
Workforce capacity	• Advocate for inclusion of dietitians in RT teams • Establish routine dietitian consultations for all high‑risk cancers • Provide basic nutrition training for RO
Nutrition screening & assessment	• Implement validated screening tools into RT workflow • Promote use of simple anthropometrics where resources are limited
Body composition assessment	• Use opportunistic CT‑based muscle assessment from routine RT planning scans • Use DXA for body composition as well as bone density • Develop simple protocols for integrating body composition into toxicity prediction
Nutrition interventions	• Develop context‑appropriate nutrition pathways for RT patients • Prioritize early oral nutrition support and counselling • Strengthen referral pathways for enteral nutrition
Patient education	• Create standardized, culturally adaptable patient education resources • Provide guidance on managing common RT‑related symptoms
Institutional infrastructure	• Develop low–cost, scalable nutrition care models • Integrate nutrition into RT quality–improvement initiatives

This study has several limitations. Convenience sampling through IAEA mailing lists and social media means the number of individuals who viewed or received the survey link is unknown, preventing calculation of a response rate and introducing potential self‑selection bias, as respondents may have been more professionally connected to IAEA activities or more interested in nutrition care. Although responses were received from 65 countries, the sample may not be fully representative of all ROs globally, and both country‑level and institutional clustering may influence the distribution of perspectives. To avoid overrepresentation, institutional practices were based on a single fully completed response per hospital, which may not capture internal variability. One UMIC contributed a disproportionately large share of responses; however, a sensitivity analysis excluding this country showed no change in the direction or significance of results, indicating that the observed income‑group patterns were not driven by this imbalance. Despite these limitations, the findings highlight the widespread recognition of nutrition as a critical component of RT care among ROs and underscores the need for coordinated global efforts to strengthen oncology nutrition capacity, expand access to evidence–based resources, and integrate body composition assessment into routine cancer care pathways.

## CRediT authorship contribution statement

**Alexia J. Murphy-Alford:** Writing – review & editing, Writing – original draft, Project administration, Methodology, Investigation, Conceptualization. **Jonathan P. Bennett:** Writing – review & editing, Writing – original draft, Methodology, Formal analysis, Conceptualization. **Aleksandra Elwertowska:** Writing – review & editing, Formal analysis, Data curation. **Elena Fidarova:** Writing – review & editing. **Aaron Grossberg:** Writing – review & editing, Writing – original draft, Methodology, Formal analysis, Conceptualization. **Deepa Puttaswamy:** Writing – review & editing, Methodology, Conceptualization. **Nuzhat Zahin:** Writing – review & editing. **Yavuz Anacak:** Writing – review & editing, Writing – original draft, Methodology, Conceptualization.

## Declaration of competing interest

The authors declare that they have no known competing financial interests or personal relationships that could have appeared to influence the work reported in this paper.
